# A macro- and micronutrient-fortified complementary food supplement reduced acute infection, improved haemoglobin and showed a dose–response effect in improving linear growth: a 12-month cluster randomised trial

**DOI:** 10.1017/jns.2019.18

**Published:** 2019-06-27

**Authors:** Shibani A. Ghosh, Nicholas R. Strutt, Gloria E. Otoo, Devika J. Suri, Judith Ankrah, Thomas Johnson, Paul Nsiah, Chie Furuta, Hitoshi Murakami, Gillian Perera, Kenneth Chui, Kennedy Bomfeh, Harold Amonoo-Kuofi, Kwaku Tano-Debrah, Ricardo Uauy

**Affiliations:** 1 Nevin Scrimshaw International Nutrition Foundation, Boston, MA, USA; 2 Friedman School of Nutrition Science and Policy, Tufts University, Boston, MA, USA; 3 University of Ghana, Legon, Accra, Ghana; 4 University of Cape Coast, Cape Coast, Ghana; 5 Ajinomoto Co. Inc., Chūō, Tokyo, Japan; 6 Pontafica University of Chile, Santiago, Chile

**Keywords:** Complementary feeding, Linear growth, Hb, KOKO Plus, AGP, α-1-acid-glycoprotein, B, baseline, CRP, C-reactive protein, E, endline, GHS, Ghana Health Service, IGF-1, insulin-like growth factor-1, KP, KOKO Plus, LAZ, length-for-age *Z*-score, M, midline, MN, micronutrient powder, MUAC, mid upper arm circumference, NE, nutrition education, RNI, recommended nutrient intakes, WAZ, weight-for-age *Z*-score, WHZ, weight-for-height *Z*-score, WLZ, weight-for-length *Z*-score

## Abstract

Inadequate protein quality may be a risk factor for poor growth. To examine the effect of a macronutrient–micronutrient supplement KOKO Plus (KP), provided to infants from 6 to 18 months of age, on linear growth, a single-blind cluster-randomised study was implemented in Ghana. A total of thirty-eight communities were randomly allocated to receive KP (fourteen communities, *n* 322), a micronutrient powder (MN, thirteen communities, *n* 329) and nutrition education (NE, eleven communities, *n* 319). A comparison group was followed cross-sectionally (*n* 303). Supplement delivery and morbidity were measured weekly and anthropometry monthly. NE education was provided monthly. Baseline, midline and endline measurements at 6, 12 and 18 months included venous blood draws, diet, anthropometry, morbidity, food security and socio-economics. Length-for-age *Z*-score (LAZ) was the primary outcome. Analyses were intent-to-treat using mixed-effects regressions adjusted for clustering, sex, age and baseline. No differences existed in mean LAZ scores at endline (−1·219 (sd 0·06) KP, −1·211 (sd 0·03) MN, −1·266 (sd 0·03) NE). Acute infection prevalence was lower in the KP than NE group (*P* = 0·043). Mean serum Hb was higher in KP infants free from acute infection (114·02 (sd 1·87) g/l) than MN (107·8 (sd 2·5) g/l; *P* = 0·047) and NE (108·8 (sd 0·99) g/l; *P* = 0·051). Compliance was 84·9 % (KP) and 87·2 % (MN) but delivery 60 %. Adjusting for delivery and compliance, LAZ score at endline was significantly higher in the KP *v.* MN group (+0·2 LAZ; *P* = 0·026). A macro- and micronutrient-fortified supplement KP reduced acute infection, improved Hb and demonstrated a dose–response effect on LAZ adjusting consumption for delivery.

Prevention of stunting in infants and children requires ‘access to’ and ‘actual intake’ of nutritious food with exclusive breastfeeding for the first 6 months of life followed by continued breastfeeding and high-quality complementary foods (e.g. animal-source foods and/or fortified complementary foods) from 6 to 24 months of age. This needs to be further coupled with access to clean drinking water and sanitation and preventive/curative health care and multiple micronutrient supplementation or fortification particularly to address deficiencies of vitamin A, Fe and Zn^(^^1^^–^^3^^)^. To date, most interventions aiming to improve linear growth have shown mixed results and current recommendations indicate the need to explore other strategies of intervention^(^^4^^,^^5^^)^.

Within the context of Ghana, a review of latest national estimates showed that while stunting rates are low at 6–8 months of age (6 %) they increased to almost 22 % at 18–23 months and 28 % by the time infants turn 24–35 months of age^(^^6^^,^^7^^)^. Furthermore, only 52 % of infants were exclusively breastfed (0–5 months of age) and 13 % achieved a minimally acceptable diet (6–23 months of age), thus indicating a severe gap in receiving optimal nutrition due to suboptimal breast feeding and complementary feeding.

While the absolute amount of protein required in early infancy and childhood is small, essential amino acid needs are significantly higher (mg/g protein)^(^^8^^)^. A strong correlation of high-quality protein availability at the national level and prevalence of stunting has been observed in an ecological analysis^(^^9^^)^. In Ghana, low protein quality adjusted for total energy was associated with an increased risk of stunting in children aged 2–13 years^(^^10^^)^, while introducing animal-source foods, which are a source of high protein quality and micronutrients, has shown a positive impact on weight gain and lean body mass in Kenyan school children^(^^11^^)^.

Protein intake in early life has been positively associated with height and weight at 10 years of age and has been indicated to have a specific growth-stimulating effect during the complementary feeding period^(^^12^^,^^13^^)^. Studies focusing specifically on individual essential and non-essential amino acids have found improved immune parameters in both adults and children^(^^14^^–^^16^^)^ as well as reduced diarrhoeal morbidity in children^(^^15^^)^. There was a significant association between low circulating levels of plasma amino acids and stunting in Malawian children under 5 years of age^(^^17^^)^ and a diet in infancy that is high in arginine and lysine was associated with better linear growth and higher fat-free mass at 10 years of age^(^^18^^)^. However, there are very few studies that have examined the potential role of protein quality coupled with micronutrient supplementation in early-life nutrition (i.e. complementary feeding) and its effect on linear growth within the first 2 years of life.

The aim and objectives of the present study were to examine the effect of providing a macro- and micronutrient-fortified complementary food supplement (formulated to improve nutritional quality of complementary foods) called KOKO Plus (KP) to infants starting at 6 months of age until 18 months of age (12-month intervention period) on change in length-for-age *Z*-score (LAZ score)^(^^19^^)^. In addition, the effect on morbidity, infection and micronutrient status was assessed. We hypothesised, all things constant, that infants from communities that received KP coupled with nutrition education (NE) would have significantly greater LAZ compared with those from communities that received a micronutrient powder with NE (MN) or NE alone. Secondary outcomes included change in weight-for-age *Z*-score (WAZ), weight-for-height *Z*-score (WHZ), mid upper arm circumference (MUAC), prevalence of anaemia, serum Hb, serum ferritin, serum Zn, serum retinol binding protein, serum cortisol, serum insulin-like growth factor-1 (IGF-1), serum C-reactive protein (CRP) and serum α-1-acid-glycoprotein (AGP), change in acute and chronic infection status and prevalence and change in the prevalence of fever and diarrhoea.

## Methods

This was a cluster randomised single-blind study with three groups (KP, MN and NE). The study design ensured participant blinding and prevented contamination. One group received KP (KP group) with NE, a second group received MN (MN group) with NE and a third group received NE alone (NE group). Due to ethical considerations, there was no control group, but we followed a separate group of communities cross-sectionally to ascertain secular trends in the primary outcome, i.e. the LAZ.

The ingredient composition and nutrient content of KP (sachet per d) are presented in Tables 1 and 2, respectively. The development of the formulation is discussed elsewhere^(^^20^^,^^21^^)^. KP contains soya powder, sugar and oil along with the essential amino acid lysine and a micronutrient premix. KP was formulated as a complement that aids meeting the WHO complementary feeding guidelines^(^^22^^)^, the FAO/WHO micronutrient recommended nutrient intakes (RNI) and the WHO protein and essential amino acid requirements for the 6- to 24-month age group^(^^8^^,^^23^^)^. It achieved 30 % of the total recommended energy requirement, 60 % of total protein and 40 % of total fat requirements from complementary foods. In addition, an assessment of amino acid and micronutrient composition shows that the supplement met 35−55 % of essential amino acids and 50–150 % RNI of micronutrient needs based on the total daily requirements. The micronutrient premix provided 50–150 % RNI in both KP and MN sachets. KP was produced in Ghana and the micronutrient premix for both supplements was produced in South Africa.

**Table 1.** Formulation of KOKO Plus (g per sachet)

Ingredients	g	%
Soyabeans	7·31	48·8
Palm oil	0·98	6·5
Sugar	5·60	37·3
Lysine	0·11	0·75
Micronutrient premix	1·00	6·7
Total	15·00	100

**Table 2.** Macro- and micronutrient composition of KOKO Plus per sachet compared with macronutrient requirements from complementary food (per d) and amino acid and micronutrient needs (per d)

	Amount	%		Requirement	% Met
Amount per sachet (g)	15·0				
Total energy					30
kcal	66·5			220	
kJ	278·2			920	
Protein (g)	2·9	18			
Utilisable protein (g)	2·6	16		4·25	62
Carbohydrate (g)	8·0	48			
Fat (g)	2·6	36		6·4	41
PUFA					
*n*-6 (18 : 2 undifferentiated)	0·9	12			
*n*-3 (18 : 3 undifferentiated)	0·1	2			
Proportion of *n*-6 to *n*-3		8·12			
Amino acid composition		mg/g protein	Amino acid score	Requirement	% Met
Tryptophan (g)	0·04	16·20	2·19	0·08	54
Threonine (g)	0·13	48·40	1·79	0·29	45
Isoleucine (g)	0·14	54·01	1·74	0·32	45
Leucine (g)	0·24	90·68	1·44	0·64	38
Lysine (g) (with added lysine)	0·31	116·59	2·24	0·55	56
Sulfur amino acids (methione + cysteine)	0·09	32·94	1·27	0·27	33
Histidine (g)	0·08	30·06	1·67	0·19	43
Valine (g)	0·15	55·60	1·32	0·43	35
Micronutrient composition*	Amount			Requirement	% Met
Vitamin A (μg retinol equivalents)	200·1			400·0	50
Folic acid (μg)	45·0			90·0	50
Niacin (mg)	3·1			6·0	52
Riboflavin (mg)	0·3			0·5	63
Thiamin (mg)	0·3			0·5	63
Vitamin B_6_ (mg)	0·3			0·5	56
Vitamin B_12_ (μg)	0·5			0·9	50
Vitamin C (mg)	30·4			30·0	101
Ca (mg)	220·3			500·0	44
Fe (mg)	7·0			11·6	60
P (mg)	154·5			100·0	154
Zn (mg)	2·4			4·1	59
Choline (mg)	62·5			45·9	136
Vitamin D (μg)	2·5			5·0	50
Vitamin E (mg)	2·7			5·0	54
Iodide (mg)	0·0			0·1	50
Vitamin K (μg)	11·0			15·0	73

* Micronutrient composition was the same for the micronutrient powder supplement.

### Ethics

The study protocol was reviewed and approved by the Institutional Review Boards of the Ghana Health Service (GHS) (Accra, Ghana) and the Noguchi Institute for Medical Research, Accra, Ghana. Written informed consent was obtained from both parents except in single parent households. A data safety monitoring board (DSMB) reviewed study outcomes on a quarterly basis. No interim analyses were planned or stopping rules defined.

### Sample size, study participants and groups

Sample size calculations were based on change in LAZ and diarrhoeal morbidity, with change in LAZ being the primary outcome. The sample size per group was 301, with thirteen clusters per group (about twenty-three participants/cluster, equal number of clusters). This would detect a 0·5 cm change in length (1·2 sd) in infants provided an energy-containing *v.* non-energy-containing micronutrient supplement using a design effect of 1·66, intraclass correlation of 0·03, power of 0·80, α of 0·05 and an attrition rate of 15 %. The sample size was also sufficient to detect a minimum of a 0·19 change (0·54 sd) in LAZ (required sample size: 298 per group). This LAZ change estimate was the average change observed by Adu-Afarwuah *et al.*^(^^24^^)^ in a three-arm intervention study with a cross-sectional non-intervention group comparing one macro- and micronutrient-fortified spread with two micronutrient formulations^(^^24^^,^^25^^)^.

**Fig. 1.**
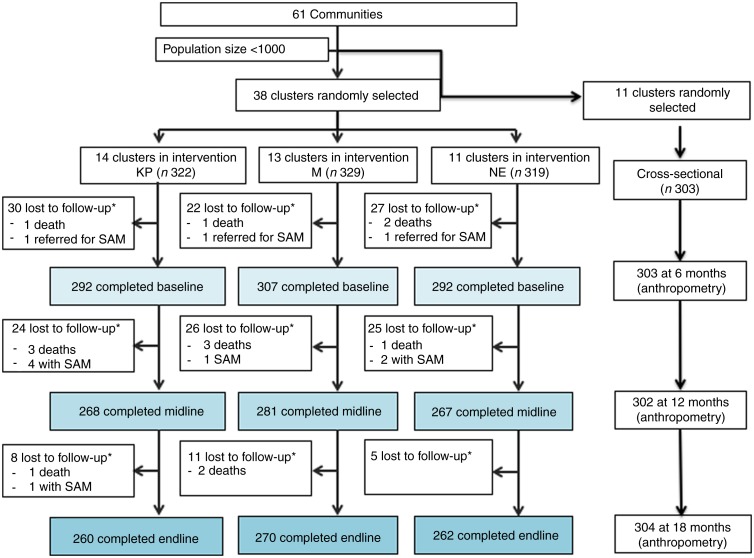
Study participants and follow-up by group (KOKO Plus (KP), micronutrient powder (MN) and nutrition education (NE)). * Lost to follow-up includes deaths and severe acute malnutrition (SAM).

The subjects were from communities in three districts of the Central region of Ghana. These were districts with the highest rates of moderate and severe acute malnutrition. A population size greater than 1000 households was defined as the minimum criterion for study inclusion. At total of sixty-one communities, each serving as a cluster, fulfilled the criterion. A total of thirty-nine communities were randomly selected using the Microsoft Excel random number function (RAND) by a research associate. Following this, a new random sequence was generated using RAND followed by block randomisation (number of blocks = 4) and the clusters were randomly assigned to one of three groups (KP, MN and NE) by the same research associate. Another eleven communities were randomly but separately selected from the remaining list. Changes occurred to the total number of clusters per group as study implementation began. During the community sensitisation process, we found one of the clusters in the KP group subdivided into two different communities and four clusters in the NE group merged into two. Thus, the total number of clusters were thirty-eight not thirty-nine, with fourteen in the KP, thirteen in the MN (original allocation) and eleven in NE group. Fig. 1 shows the actual study flow, loss to follow-up and ‘drop outs’ by individual participants. The study was conducted from January 2013 through to February 2015 when the last infant graduated from the intervention study.

### The intervention

The University of Ghana implemented the study. The intervention period was 12 months from infant age 6−18 months and was delivered at the community level to ensure blinding and prevent contamination. The KP and MN supplements were formulated for daily consumption with instructions for use to mothers in the communities assigned to KP and MN groups, respectively. The distribution was conducted by a local non-governmental organisation (NGO) working with community health volunteers. The distribution team was expected to visit each community on a weekly basis and deliver the supplements through the community health volunteers. All the mothers enrolled in the study were followed by the community health volunteers who live in the communities. The NE materials were adapted from the Good Life project, a US Agency for International Development behaviour change project conducted from 2009 to 2013, to support GHS in areas of family planning, maternal and child health, malaria, nutrition, water and sanitation. Modifications were made to the training materials with specific modules on supplement use^(^^26^^)^. The NE component included monthly sessions with mothers and infants with role-plays, activities and cooking demonstrations conducted in conjunction with GHS volunteers in each community irrespective of treatment group.

In each community irrespective of intervention group, all mothers with newborn infants (0–3 months of age) who attended the mother support group were invited to participate in the study. This was to encourage mothers to participate in monthly nutrition education sessions and to continue exclusive breastfeeding. When eligible (at 6 months of age), dyads were enrolled into the intervention. Inclusion criteria were singleton term birth, exclusively or predominantly breastfed, parents planning to live in the community for a period of 12 months and willing to participate for the entire study period and written informed consent. Exclusion criteria included severe anaemia (Hb <70 g/l) or severe acute malnutrition (MUAC <110 mm)^(^^27^^,^^28^^)^. Infants were assessed for severe anaemia and acute malnutrition at each time point (baseline (B), midline (M) and endline (E)) and, if diagnosed, referred for routine medical care and excluded from participation.

### Anthropometric measurements

Anthropometric measurements were collected monthly and included length (Infant/Child ShorrBoard^®^; Weigh and Measure, LLC; http://www.weighandmeasure.com/), weight (Seca 874 digital scale; http://www.seca.com/en_mw/products/all-products/product-details/seca874.html), MUAC (Child MUAC Tape; Weigh and Measure, LLC), subscapular and triceps skinfolds (Holtain T/W skinfold caliper; http://www.holtain.com/tw.php) and head and chest circumference. The digital scales were tested weekly for accuracy using standard weights. Motor development assessments were conducted using the WHO motor development skills framework^(^^29^^)^.

### Questionnaires

A single 24-h diet recall and semi-structured questionnaires were administered to assess change in diet, socio-economic status, infant and young child feeding practices, morbidity and household food security at B, M and E. The 24-h diet recall was developed and implemented in different studies within the University of Ghana and was contextualised to the local diet. A series of locally tested and validated household measures were used for ascertaining portion sizes. Dietary data were cleaned with all data in household measures converted into grams. Supplement compliance and morbidity questionnaires were administered weekly. Both paper and electronic forms were utilised. Data were uploaded daily through the cell phone network, stored on Formhub and ONA (the Formhub system began having problems in mid-2014 and stopped being maintained by developers. ONA is an identical system, which made the switch seamless). All data cleaning and analysis was done using Stata 13.1 (StataCorp LLC).

### Clinical measures and sample handling

One venous blood draw (3 ml) and a fingerprick (Hemocue 301), to assess severe anaemia (<70 g/l) (for screening purposes), were collected at B, M and E^(^^30^^)^. Sample collection utilised butterfly needles (21/23 gauge) and K_2_EDTA vacutainers (catalogue no. 368841; Becton, Dickinson and Company; https://www.bd.com/resource.aspx?IDX=7220) for whole blood and plasma analyses and Trace Element Serum Separator Tube vacutainers (BD; catalogue no. 368380) for serum analyses. Samples were immediately placed in a super cooler tube rack to keep sample temperature at 4°C^(^^31^^)^ and transported back to the laboratory within 5 h of collection.

A quantity of 50 μl of K_2_EDTA blood was aliquoted into an Eppendorf tube for whole blood count including Hb concentration (ABX Pentra-60 haematology machine; Horiba Medical; http://www.horiba.com/us/en/medical/products/hematology/abx-pentra-60/). Hb concentrations measured clinically were used for subsequent statistical analyses while Hb measured with HemoCue were used for screening infants. The rest was centrifuged at 3000 ***g*** (14 min at 4°C) and plasma aliquoted into Eppendorf tubes and stored at −20°C for plasma amino acid analysis (to be reported in a separate paper). Samples collected in trace element-free tubes were rested at room temperature for 30 min, centrifuged at 3000 ***g*** (15 min, 25°C), and aliquoted using sterile metal-free pipette tips (200 µl) into one DNase, pyrogen-free microcentrifuge tube (Thomas Scientific) stored at −80°C for Zn analysis and three Eppendorf tubes stored at −30°C. Serum Zn (Elemental Analysis Lab, David Killilea, University of California) was measured using HPLC, IGF-1, serum cortisol and prealbumin using ELISA (IGF-1 and Cortisol kits from DRG; Prealbumin kit from Abcam) (University of Cape Coast, Ghana), serum transferrin receptors, serum retinol binding protein, serum ferritin, CRP and AGP using sandwich ELISA (Juergen Erhardt)^(^^32^^)^.

### Statistical analysis

The primary outcome of this study was change in mean LAZ from 6 to 18 months of age in infants of the KP group compared with those of the MN and NE groups. Anthropometric indices (LAZ, WAZ and weight-for-length *Z*-score (WLZ)) were computed using the WHO 2006 growth reference charts (WHO macro, STATA)^(^^33^^)^ as were the household insecurity access score (HFIAS)^(^^34^^)^, maternal BMI and dietary diversity scores^(^^35^^)^.

Duplicate anthropometric measurements were checked for discrepancies. A total of six observations were flagged as implausible *Z*-scores by WHO 2006 definitions and were coded as missing. Outlier tests on an infant's longitudinal measurements were conducted using a linear regression model which computed standardised residuals for each data point. A standardised residual ≥ +2·5 or ≤ −2·5 was considered as an outlier and replaced through imputation. A new regression was run and used to impute the omitted length measurement with a new predicted value. This process was repeated using a less strict cut-off of ≥ +3·0 or ≤ −3·0 standardised residual. Based on the review of the findings, the study statistician recommended using the more stringent cut-off of ≥ +2·5 or ≤ −2·5. Across all the anthropometric data and intervention groups (*n* 9161 observations, including measurements at baseline (B), midline (M) and endline (E) and monthly measurements), a total of seventy-six data points were identified as outliers and replaced through imputation.

All analyses were intent to treat. To verify the randomisation assumption, differences in mean values across three groups at B were tested using linear mixed-effects regression models adjusting for clustering. The difference in difference across groups and intervention period was tested using mixed-effects linear regression models adjusting for clustering, intervention group, B values and repeated measures. For all models (both primary and secondary outcomes), random effects included in the model were the cluster variable and the individual ID while baseline and demographic variables (determined using step-wise regression and vary by each model) were included as fixed effects. Any additional covariates in the models were included based on the specific outcome measure. The primary outcome was LAZ. Secondary outcomes included WAZ, WLZ, MUAC, serum Hb, anaemia prevalence, serum ferritin (unadjusted and adjusted for inflammation), serum Zn, serum cortisol, serum IGF-1, serum retinol binding protein, serum CRP and serum AGP, acute and chronic infection status and prevalence, and prevalence of fever and diarrhoea.

For LAZ and all other anthropometric outcomes, we computed two separate models. One is the B-E model, where we estimated changes from B (6 months) to M (12 months) and B to E (18 months of age) using the B, M and E data for anthropometry. The second is the monthly model where we computed marginal treatment effects over an 18-month period to examine the rate of change in LAZ, WAZ and WLZ using the monthly anthropometric data. Separately to assess secular differences between the three arms and the comparison group, we computed LAZ, WAZ and WHZ scores for each of the time points (6, 12 and 18 months) and examined change at E adjusting for B, age, sex and clustering using mixed-effects models.

We used the two inflammation markers CRP and AGP to determine prevalence of acute and chronic inflammation. Onset of acute inflammation was defined as CRP >5 mg/l while presence of chronic inflammation was defined as AGP >1 g/l as defined and utilised by several studies^(^^36^^–^^40^^)^. Prevalence of acute and chronic inflammation was computed for B, M and E and change in prevalence was tested using mixed-effects logistic regression analysis. We further utilised the CRP and AGP data and above-noted cut-offs to compute a variable for the four infection stages as defined by Thurnham *et al.*^(^^39^^)^. For the biochemical markers, models were adjusted for age, sex, B value, community clustering and the infection stage^(^^39^^)^. We tested two approaches with serum ferritin: one where the unadjusted serum ferritin was modelled with infection stage (at the different time points) as a covariate and a second where the serum ferritin itself was adjusted. For Hb, we examined the difference across groups between children with or without acute infection at E, adjusting for chronic infection and sex, given the high risk of malaria in this population^(^^41^^,^^42^^)^. Serum ferritin at each time point was adjusted for inflammation at that time point using the four-stages method as recommended by Thurnham *et al.*^(^^39^^)^.

For the morbidity markers, while data on presence or absence of malarial parasite was not collected, we assessed morbidity using two common measures: prevalence of fevers (all fevers including malarial) and diarrhoeal episodes at B, M and E, again using mixed-effects models. Diarrhoea is defined as three or more loose or liquid stools per d. Episodes of diarrhoea are considered separate if there are three or more consecutive diarrhoea-free days. Prevalence of fever was also assessed at B, M and E as presence of any fever as reported by the caregiver in the past week. While one of the outcome measures was to examine diarrhoeal morbidity using the longitudinal (weekly) data, we were unable to compute these indicators due to significant missing data and thus report only on the B-M-E changes in both acute infection and diarrhoeal morbidity using mixed-effects logistic regression models.

Nutrient intake analysis and dietary diversity score computations were conducted. Nutrient intake was calculated using the Research to Improve Infant Nutrition and Growth (RIING) food composition table of Ghanaian foods compiled from three different data sources including FAO, US Department of Agriculture (USDA) and data used in previous research in Ghana (RIING food composition database, Nutrition Department, University of Ghana). The database contains 306 foods with twenty-nine nutrients (macro- and micronutrients). Nutrient intakes were calculated using a SAS (version 9.3; SAS Institute) program. Mean intakes and corresponding standard deviations were estimated at B, M and E. Differences were tested using ANOVA.

Compliance as defined by total used sachets divided by total delivered sachets was 86·2 % in the KP group and 88·4 % in the MN groups, indicating that if the mother received the sachet, the sachets were utilised at a similar rate across the two groups (Table 3). However, mean consumption (total used sachets) across both groups was 186 supplements (181 in KP, 190 in MN), much lower than the expected 365 supplements over 52 weeks. Thus, while compliance was high, this was a measure only of the total received supplements. A review of the delivery and distribution logs showed that, on average, mothers received only about two-thirds of the expected 365 supplements. Supplement delivery was hampered due to various reasons – including inaccessibility to mothers, inaccessibility to sites during the monsoons among others.

**Table 3.** Delivery and reported consumption of supplement and overall compliance during the trial period, in the KOKO Plus and micronutrient groups (Mean values and standard deviations; medians and percentages)

	KOKO Plus				Micronutrients			
Supplement exposure	Mean	sd	Median	%	Mean	sd	Median	%
Number of supplements delivered to subjects	225·1	77·6	238	–	225·9	79·5	231	–
Proportion of intended delivery (365 supplements) (%)	–	–	65·2	61·7	–	–	63·3	61·9
Number of supplements reportedly consumed	196·1	81·9	207	–	201·9	81·7	201	–
Percentage compliance (number of supplements consumed as a percentage of those delivered)	–	–	93·3	86·2	–	–	93·6	88·4

Supplement consumption, i.e. study adherence, was thus affected by delivery. As high rates of non-adherence can lead to underestimates of treatment effect^(^^43^^)^, we examined the relationship of supplement consumption and each outcome measure (both primary and secondary). As short-term variation in supplement consumption (i.e. 1 week or 1 month) would not be observable, we included a variable that represents total supplements consumed by the child over the duration of the study. As the total supplement consumed by an infant over the study would have had a different effect on outcomes at M compared with E, this variable was modelled as an interaction with time and intervention/treatment group. The interaction term of time, treatment and consumption allowed for the effect of supplement consumption (a time-invariant variable) to be modelled differentially over time. Thus, we conducted consumption modelling using mixed-effects regression models and we estimated predicted outcomes at different time points across a range of different levels of supplement consumption which represented adjusted means over time at those different levels.

## Results

A total of 970 infants were recruited, 891 completed B, 816 completed M and 792 completed the E measurements (Fig. 1). Following the B measurement, thirty-two children in the KP group (twenty-four before E, eight before E), thirty-seven children in the MN group (twenty-six before M, eleven before E) and thirty children in the NE group (twenty-five before M and five before E) dropped out or were lost to follow up due to maternal refusal, moving out of the area, severe anaemia, infant deaths and severe acute malnutrition (Fig. 1). All deaths and referrals were reported to the Noguchi Institute for Medical Research Institutional Review Board and the study data safety monitoring board (DSMB); none was found to be related to the study.

### Baseline characteristics

The age of male and female infants was comparable across groups. The rate of exclusive breastfeeding through 6 months of age was 41 % in the NE and 51 % in the KP group, but the difference in breastfeeding did not reach a level of statistical difference (Table 4). The LAZ score in the NE group was lower than those observed in the KP and the MN groups, but the differences were not significant (Table 5).

**Table 4.** Baseline descriptive characteristics across the KOKO Plus, micronutrient and nutrition education groups* (Percentages and frequencies; mean values and standard deviations)

	KOKO Plus			Micronutrients			Nutrition education		
	Sample size (*n*)	%	Frequency	Sample size (*n*)	%	Frequency	Sample size (*n*)	%	Frequency
Number of clusters			14			12			11
Infant characteristics									
Infant given Fe supplement	187	26	49	185	25	46	182	33	60
Use of iodised salt for infant's food	141	39	55	158	33	52	171	33	57
Female (sex of infant)	292	51	150	307	53	163	292	48	141
Exclusive breastfeeding through 6 months of age	286	51	145	301	48	143	291	41	119
Infant receiving vitamin A supplement	190	83	158	193	79	152	200	84	168
Age of infant at recruitment (months)	287			301			289		
Mean		2·32			1·97			1·89	
sd		1·64			1·59			1·26	
Age of infant at baseline (months)	287			301			289		
Mean		6·11			6·18			6·21	
sd		0·62			0·50			0·72	
Maternal characteristics									
Maternal education (none)	287	13	38	301	18	53	289	14	41
Primary school completion (mother)	287	77	220	301	66	198	289	70	203
Total children <5	287			303			290		
Mean		1·61			1·67			1·61	
sd		0·74			0·71			0·77	
Number of children alive	287			303			291		
Mean		2·89			3·16			2·84	
sd		2·00			2·02			1·72	
Number of children born	287			303			291		
Mean		3·03			3·32			2·98	
sd		2·23			2·17			1·88	
Age of mother at first birth (years)	287			303			291		
Mean		20·24			20·05			20·08	
sd		3·68			3·36			3·61	
Maternal BMI (kg/m^2^)	274			288			272		
Mean		23·35			23·26			23·15	
sd		3·91			3·97			3·95	
Household characteristics									
Land ownership	287	56	160	303	61	184	291	48	141
Improved toilet	287	80	231	303	79	240	291	75	218
Presence of electricity	287	84	241	303	76	231	290	83	240
Improved water source	287	94	271	303	94	285	291	83	242
Household food security score	287			301			290		
Mean		5·02			5·78			6·45	
sd		4·68			5·09			5·00	

* No significant differences in characteristics at baseline.

**Table 5.** Baseline anthropometry, biochemistry and morbidity markers (Percentages and frequencies; mean values and standard deviations)

	KOKO Plus			Micronutrients			Nutrition education		
	Sample size	%	*n*	Sample size	%	*n*	Sample size	%	*n*
Anthropometric markers									
LAZ	286			300			288		
Mean		−0·74			−0·74			−0·64	
sd		1·02			0·97			1·01	
WAZ	286			299			288		
Mean		−0·71			−0·69			−0·69	
sd		1·14			1·10			1·12	
WLZ	286			299			287		
Mean		−0·23			−0·24			−0·32	
sd		1·14			1·02			1·12	
Biochemical markers									
Hb (g/l)	266			286			269		
Mean		112·92			113·88			113·17	
sd		25·39			20·78			22·06	
% Anaemia (Hb ≤109 g/l)	266	44	118	286	41	116	269	41	108
% Mild anaemia (Hb 100–109 g/l)	266	23	61	286	22	64	269	18	48
% Moderate anaemia (Hb 70–99 g/l)	266	18	48	286	17	48	269	21	56
% Severe anaemia (Hb ≤ 70 g/l)	266	3	9	286	1	4	269	2	7
Ferritin (μg/l)	280			294			276		
Mean		40·13			50·03			44·58	
sd		50·66			47·55			47·36	
Inflammation-adjusted ferritin (μg/l)	280			294			276		
Mean		41·2			41·6			37·1	
sd		35·7			37·8			35·8	
% with low ferritin (<12 µg/l)	280	20	56	294	14	40	276	21	57
Retinol binding protein (μmol/l)	280			294			276		
Mean		0·97			0·97			0·97	
sd		0·25			0·25			0·33	
Zn (μg/ml)	101			101			101		
Mean		0·64			0·62			0·63	
sd		0·16			0·15			0·15	
Growth markers									
IGF-1 (ng/ml)	279			295			276		
Mean		63·82			59·81			65·46	
sd		33·72			36·23			32·89	
Cortisol (ng/ml)	266			288			269		
Mean		65·82			65·00			64·67	
sd		36·65			33·99			34·77	
Inflammation markers									
CRP (mg/l)	280			294			276		
Mean		3·92			3·31			3·27	
sd		10·38			7·20			7·29	
% Acute inflammation (CRP >5 mg/l)	280	16	45	294	16	47	276	17	48
α-Glycoprotein (g/l)	280			294			276		
Mean		0·90			0·91			0·92	
sd		0·69			0·57			0·65	
% Chronic inflammation (AGP >1 g/l)	280	25	69	294	28	82	276	27	75
Infection stage	292			307			294		
None (CRP−, AGP−)		68			66			65	
Incubation (CRP+, AGP−)		4			3			3	
Early convalescence (CRP+, AGP+)		11			13			13	
Late convalescence (CRP–, AGP+)		12			14			12	
Morbidity markers									
Prevalence of diarrhoea (7 d)		6·60			4·40			11·50	
Prevalence of fever (7 d)	287	30		300	26		278	33	

LAZ, length-for-age *Z*-score; WAZ, weight-for-age *Z*-score; WLZ, weight-for-length *Z*-score; IGF-1, insulin-like growth factor-1; CRP, C-reactive protein; AGP, α-1-acid-glycoprotein.

### Differences in length-for-age *Z*-score, weight-for-age *Z*-score and weight-for-length *Z*-score across groups

**Fig. 2.**
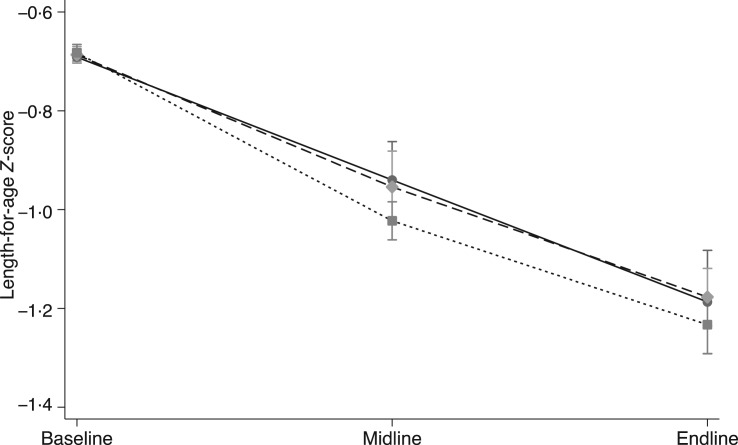
Mean length-for-age *Z*-scores (LAZ) of children at baseline, midline and endline, by group: –●–, KOKO Plus; 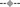, micronutrient powder; 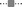, nutrition education. We used a mixed-effects linear model adjusted for fixed effects of baseline LAZ and mother's height, and random effects of study cluster and subject to account for repeated measures.

The B-E and monthly models were slightly different, but no significant differences were found in the change in LAZ (Fig. 2), WAZ or WLZ scores using either model (Table 6). The data for the three intervention groups (B, M and E time points) were also compared with the growth monitoring cross-sectional group. Adjusting for B values, no significant differences were found at any time point between the three intervention groups and the cross-sectional group (data not shown).

**Table 6.** Effect of KOKO Plus on anthropometric outcomes (monthly model *v.* baseline–endline (B-E) model)* (Mean values with their standard errors; mean differences and 95 % confidence intervals)

		KOKO Plus		Micronutrients					Nutrition education				
	*n*	Mean	se	Mean	se	Difference	95 % CI	*P*	Mean	se	Difference	95 % CI	*P*
Estimated outcomes at endline (monthly model; 18 months)													
LAZ	8679	−1·219	0·064	−1·211	0·031	0·008	−0·132, 0·147	0·912	−1·266	0·031	−0·047	−0·187, 0·092	0·505
WLZ	8590	−0·625	0·06	−0·546	0·026	0·079	−0·049, 0·206	0·225	−0·658	0·065	−0·033	−0·206, 0·139	0·705
WAZ	8622	−0·976	0·059	−0·947	0·03	0·029	−0·101, 0·158	0·663	−1·055	0·047	−0·079	−0·226, 0·068	0·29
Estimated outcomes at endline (B-E model)													
LAZ	2357	−1·187	0·053	−1·177	0·03	0·01	−0·11, 0·13	0·87	−1·233	0·03	−0·046	−0·166, 0·074	0·451
WLZ	2330	−0·521	0·068	−0·434	0·025	0·087	−0·055, 0·229	0·23	−0·532	0·065	−0·011	−0·195, 0·173	0·908
WAZ	2335	−0·934	0·062	−0·899	0·025	0·035	−0·096, 0·165	0·603	−0·994	0·041	−0·06	−0·205, 0·085	0·421

LAZ, length-for-age *Z*-score; WLZ, weight-for-length *Z*-score; WAZ, weight-for-age *Z*-score.

* Mixed-effects linear regression models controlling for baseline value of outcome, age, sex, mother's height (LAZ models), mother's BMI (WHZ and WAZ models) and community clustering.

### Differences in micronutrient and growth markers across groups

Hb levels at E were higher in the KP group compared with the levels found in the MN and the NE groups, but the differences were not significant (Table 7). Adjusting for B value, sex, age and clustering, serum retinol binding protein, serum Zn, serum cortisol and IGF-1 were higher at E in the KP compared with the NE group but these differences were not statistically significant. Adjusting for B value, sex, age and clustering, serum retinol binding protein, serum Zn and serum IGF-1 at E were higher in the KP group than the MN group but serum ferritin and serum cortisol were lower (Table 7). None of the differences between groups for these biomarkers was significant.

**Table 7.** Effect of KOKO Plus on micronutrient and growth markers* (Mean values with their standard errors; mean differences and 95 % confidence intervals)

		KOKO Plus		Micronutrients					Nutrition education				
	*n*	Mean	se	Mean	se	Difference	95 % CI	*P*	Mean	se	Difference	95 % CI	*P*
Micronutrient biomarkers (estimated outcomes at endline)													
Hb (g/l)	2178	111·81	1·904	107·012	2·638	−4·796	−11·194, 1·601	0·142	108·664	0·813	–3.144	–7.183, 0.895	0·127
Unadjusted ferritin (μg/l)	2295	43·52	2·504	45·457	3·52	1·936	−6·575, 10·447	0·656	39·705	2·814	–3.817	–11.327, 3.694	0·319
Retinol binding protein (μmol/l)	2298	0·96	0·017	0·946	0·016	−0·013	−0·059, 0·032	0·567	0·939	0·01	0.939	0.01	0·327
Zn (μg/ml)	900	0·60	0·022	0·577	0·011	−0·023	−0·07, 0·023	0·567	0·582	0·01	–0.019	–0.065, 0.027	0·427
Growth biomarkers (estimated outcomes at endline)												
IGF-1 (ng/ml)	2295	39·08	1·261	38·813	2·334	-0·263	−5·465, 4·94	0·921	36·842	1·139	–2.234	–5.58, 1.113	0·191
Cortisol (ng/ml)	2219	79·14	2·936	82·211	3·082	3·077	−5·397, 11·55	0·477	74·873	2·309	–4.262	–11.804, 3.28	0·268

IGF-1, insulin-like growth factor-1.

* Mixed-effects linear regression models controlling for baseline value of outcome, age, sex, infection stage (composite of α-1-acid-glycoprotein and C-reactive protein values) and community clustering.

### Differences in inflammation and inflammation-adjusted biomarkers and morbidity across groups

At E, serum CRP and AGP levels were lower in the KP group when compared with the findings in other groups, but the differences did not reach a level of significance. Prevalence of acute inflammation was also lower in the KP than the MN (*P* = 0·043) and NE groups (*P* > 0·05). There was no difference in serum ferritin, whether unadjusted (Table 7) or adjusted for the infection stages using Thurnham's method (Table 8). Adjusting for chronic inflammation, KP infants with no acute inflammation had significantly higher serum Hb compared with MN infants with no acute inflammation (*P* = 0·043). The same comparison between KP and NE infants was trending towards significance (*P* = 0·051) (Table 8). The change in prevalence of fever and diarrhoea from B to E had a downward trend but was not significant (data not shown).

**Table 8.** Effect of KOKO Plus on inflammation and markers adjusted for inflammation (Mean values with their standard errors; mean differences and 95 % confidence intervals)

		KOKO Plus		Micronutrients					Nutrition education				
	*n*	Mean	se	Mean	se	Difference	95 % CI	*P*	Mean	se	Difference	95 % CI	*P*
Inflammation and infection prevalence (endline outcomes)													
CRP (mg/l)†	2345	5·02	0·666	6·18	0·682	1·161	−0·701, 3·023	0·222	6·073	0·672	1·055	−0·802, 2·912	0·266
Acute infection, prevalence (%)‡	2345	0·18	0·031	0·215	0·018	0·035	−0·031, 0·101	0·298	0·249	0·021	0·069*	0·002, 0·136	0·043
AGP (g/l)†	2345	1·17	0·052	1·18	0·032	0·014	−0·104, 0·132	0·815	1·229	0·039	0·063	−0·064, 0·19	0·332
Chronic infection, prevalence (%)‡	2345	0·43	0·043	0·445	0·028	0·017	−0·081, 0·116	0·731	0·462	0·032	0·035	−0·071, 0·14	0·519
Adjusted serum markers (endline outcomes)													
Adjusted ferritin (μg/l)§	2298	34·52	1·712	35·816	2·827	1·295	−5·176, 7·766	0·695	30·649	2·192	−3·873	−9·333, 1·587	0·164
Adjusted Hb (g/l)ǁ	2178												
No acute infection		114·02	1·868	107·791	2·516	−6·228*	−12·367, −0·089	0·047	109·878	0·995	−4·14	−8·293, 0·012	0·051
With acute infection		102·18	3·403	102·853	3·506	0·672	−8·912, 10·257	0·891	103·901	2·099	1·72	−6·134, 9·575	0·668

CRP, C-reactive protein; AGP, α-1-acid-glycoprotein.

*P < 0·05.

† CRP and AGP are adjusted for age, sex and community clustering.

‡ Acute infection prevalence and chronic infection prevalence are modelled using mixed-effects logistic regression models, and adjusted for age, sex and community clustering.

§ Ferritin was adjusted using the method described by Thurnham *et al.*^(39)^ and tested using a mixed-effects linear regression model controlling for baseline value of outcome, age, sex and community clustering.

ǁ Hb adjusted for acute and chronic infection, age, sex and community clustering with an interaction term for CRP (acute infection) by study group and time point.

### Supplement consumption and primary and secondary outcome measures

**Fig. 3.**
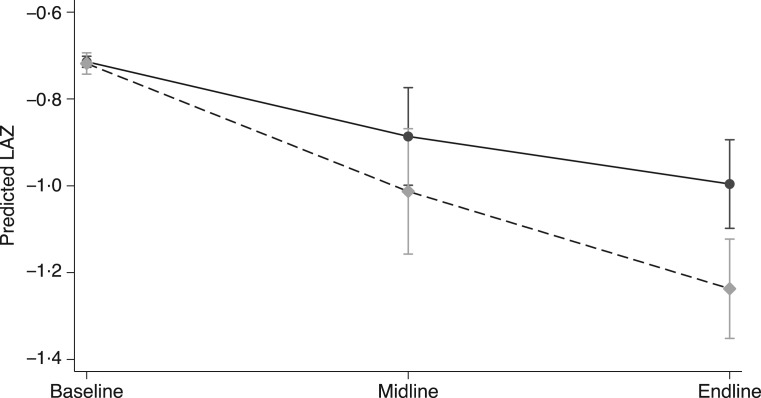
Predicted length-for-age *Z*-scores (LAZ) of children in KOKO Plus (–●–) *v.* micronutrient powder (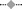) groups at baseline, midline and endline (314 supplements consumed). We used a mixed-effects linear model adjusted for fixed effects of baseline LAZ and mother's height, and random effects of study cluster and subject to account for repeated measures. Mean compliance was 86 %; for this model it was assumed that if all 365 supplements were delivered average consumption would be 314 sachets (86 % of intended delivery).

Average compliance defined by total used sachets divided by total delivered sachets was 86·2 % in the KP group, and 88·4 % in the MN group, indicating similar rate of use across groups if the mother received the sachets (Table 3). Assuming that the compliance rate remained constant, if the mothers had received supplements across all 52 weeks (365 sachets), the mean consumption across both groups would have been 314 sachets (310 sachets in the KP and 318 sachets in the MN groups). Using this calculation, we modelled the change in outcomes by difference in consumption levels between the two groups. The predicted LAZ adjusted for compliance and consumption showed a significant difference at E, with the KP group exhibiting a significantly higher LAZ (B adjusted) compared with that of the MN group (*P* = 0·002) (Fig. 3). At consumption levels of 314 sachets (the estimated compliance rate) or 365 sachets (daily consumption over a 12-month period), the infants in the KP group have a significantly higher LAZ scores than those estimated in the MN group (*P* = 0·0017) (Supplementary Fig. S1).

Hb levels were higher when the sachet intake was increased from fifty to 365 sachets in the KP group (108−116 g/l), as compared with the estimates in the MN group (106−108 g/l). Adjusting for acute infection as an interaction term, the difference between the KP and MN groups reached a significant level only at intake of 100 sachets (*P* = 0·049) and not at lower or higher levels of consumption (Supplementary Fig. S2). Serum Zn levels were higher when the sachet intake was increased from fifty to 365 sachets in the KP group (0·52−0·70 µg/ml), as compared with the estimates in the MN group (0·56−0·58 µg/ml). The difference was favouring the KP intervention group at higher consumption levels (significant only at 318 sachets/year, *P* = 0·020 and 365 sachets/year *P* = 0·017) (Supplementary Fig. S3).

## Discussion

We examined the effect on linear growth, infection and micronutrient markers of the provision of a macro- and micronutrient supplement called KOKO Plus added to the diets of Ghanaian infants starting at 6 months of age through to 18 months of age (12-month intervention) (KP group). We utilised mixed-effect regression models to examine the change in primary and secondary outcome measures.

We hypothesised that the provision of a macro- and micronutrient supplement that is enhanced for protein quality with amino acids and met micronutrient needs coupled with nutrition education would improve linear growth, reduce infection and improve micronutrient status compared with the provision of micronutrients alone or provision of nutrition education alone. The primary outcome of this study was LAZ. In intent-to-treat analyses, there were no group differences in the LAZ score at the end of the intervention, with the LAZ declining comparably in all groups. The lack of KP effect on the LAZ score contradicted previous studies showing that complementary food supplements and/or foods themselves provided for 12 months in a conjunction with nutrition education increased the LAZ score in children under 2 years of age irrespective of their food security status^(^^44^^–^^46^^)^. We assessed compliance to the intervention and ascertained an effect of delivery of the supplement on our primary outcome. Consumption modelling showed that infants in the KP group who received the expected number of sachets had significantly better LAZ scores than the MN group, indicating a dose–response effect of supplement consumption and underscores the importance of meeting the daily requirements on a consistent basis through the first 2 years of life.

Two other findings of the study, that have clinical importance, were, (A) a significant decline in the prevalence of acute infection^(^^41^^,^^47^^)^ and, (B) a significant increase in serum Hb in children without acute infection that received KP but not a significant increase in serum ferritin in the KP group. Hb levels have been shown to drop in children with acute infection^(^^41^^,^^47^^)^. The decline in acute infection and change in serum Hb but not serum ferritin implies an infection effect. On the other hand, the MN group did see an improvement in serum ferritin (adjusted) but this was not sufficiently different from the KP group. This could imply that within the context of this population, provision of 50 % RDA for Fe was insufficient to change Fe status. On a mechanistic level, it may be hypothesised that the Hb improvement was linked to the presence of lysine and/or other amino acids in KP, rather than to a non-specific improvement in Fe status, given the lack of effect on serum ferritin. It could also be further speculated that this may be associated with inflammation. Hussain *et al.*^(^^14^^)^ have documented increases in Hb levels in Pakistani women supplemented with lysine alone (with no added Fe) and an increased risk of anaemia was reported in cases of lysinuric protein intolerance^(^^48^^)^. A proposed mechanism for the effect of lysine could be through lowered levels of serum CRP, a finding observed in Ghanaian women supplemented for 16 weeks with lysine alone^(^^16^^)^. Further work needs to be conducted to elucidate the effect of individual amino acids on inflammation and markers of Fe status.

It could be argued that the lack of effect on LAZ could be due to inconsistencies in total energy intake over time across groups, inadequate breast milk intake and/or substitution and lack of delivery. Further, KP was a complementary food supplement to be used with existing porridge-type foods, while previous studies focused on complementary foods as such, allowing for a better control of energy intake^(^^49^^,^^50^^)^. However, we find that while dietary diversity did increase from B to M to E, this increase was similar across all three groups (no significant difference in change in mean dietary diversity). We also found that total energy from the diet increased from B, M and E across all three groups in an equivalent manner (there were no significant differences across groups at the same time point and no significant difference in increase over time across groups, data not shown). In addition, energy contributions from the diet at E were 622·9 (sd 370·8) kcal (2606·2 (sd 1551·4) kJ) (combined across three groups, no significant differences between groups) and the proposed recommended energy from complementary foods for the age range of 12–23 months being 548 kcal (2293 kJ)^(^^22^^,^^51^^)^. It is possible that breast milk consumption was inadequate, and the diets enumerated are not usual intake. An analysis of breast milk consumption frequency indicates that, irrespective of group assignment, infants continued to be breast fed through the intervention period. There was no variation in frequency by group and thus would not account for any differences (or lack of) in the models.

Rather than inconsistencies in total energy intake and breast feeding, the poor delivery of the supplements might have been the decisive factor negatively influencing the final LAZ score^(^^43^^)^. We used consumption modelling to examine the change in the outcomes, under conditions of optimal delivery and compliance (compliance was high at 86 % irrespective of group allocation). With those assumptions, the LAZ, WAZ and WHZ scores significantly increased at the end point in the KP but not in the MN group. Other studies have found similar patterns of improvement after accounting for consumption^(^^52^^)^. Nevertheless, the generalisability of these findings is limited, and one cannot fully attribute the lack of effect on LAZ to the poor delivery of KP supplements due to inability to compare with the NE intervention group, which did not have similar monitoring data. Finally, interpretation of serum Zn was confounded by extensive haemolysis in the samples and while new recommendations suggest an adjustment is needed, currently there is no standard procedure^(^^53^^)^. To prevent bias in the level of haemolysis by group type, we determined percentage samples haemolysed by group and found no significant difference.

In conclusion, a 12-month intervention of a fortified complementary food supplement, provided to improve nutritional quality of complementary foods, improved Hb levels in children with no acute infection, an effect that is modulated potentially by the protein quality of the supplement, possibly the amino acid lysine. The intent-to-treat analysis did not find a significant effect on LAZ; however, this is confounded not by compliance but by delivery of the supplement. An exploration of the data revealed a potential dose–response effect which was explored using consumption modelling. Using intended delivery rates with observed compliance showed a significantly higher LAZ, WAZ and WLZ at E in the KP group, confirming the dose–response effect of the intervention. This indicates a potential role of supplements like KOKO Plus in improving linear growth, acute infection and Hb and suggests that supplementation during complementary feeding is likely to achieve linear growth improvements if the supplement is consumed such as to meet the daily requirements. The findings warrant further investigation with well-controlled delivery plans.

## Supplementary material

For supplementary material accompanying this paper visit https://doi.org/10.1017/jns.2019.18. click here to view supplementary material

## References

[1] de Pee S & Bloem MW (2009) Current and potential role of specially formulated foods and food supplements for preventing malnutrition among 6- to 23-month-old children and for treating moderate malnutrition among 6- to 59-month-old children. Food Nutr Bull 30, S434–S461. 19998866 10.1177/15648265090303S305

[2] Pan American Health Organization (2003) *Guiding Principles for Complementary Feeding of the Breastfed Child* Washington, DC: Pan American Health Organization https://www.who.int/nutrition/publications/guiding_principles_compfeeding_breastfed.pdf

[3] De-Regil LM, Suchdev PS, Vist GE, (2013) Home fortification of foods with multiple micronutrient powders for health and nutrition in children under two years of age (review). Evid Based Child Health 8, 112–201. 23878126 10.1002/ebch.1895

[4] Ramakrishnan U, Aburto N, McCabe G, (2004) Multimicronutrient interventions but not vitamin A or iron interventions alone improve child growth: results of 3 meta-analyses. J Nutr 134, 2592–2602. 15465753 10.1093/jn/134.10.2592

[5] Ramakrishnan U, Nguyen P & Martorell R (2009) Effects of micronutrients on growth of children under 5 y of age: meta-analyses of single and multiple nutrient interventions. Am J Clin Nutr 89, 191–203. 19056559 10.3945/ajcn.2008.26862

[6] Ghana Statistical Service (GSS), Ghana Health Service (GHS) & ICF Macro (2009) Ghana Demographic and Health Survey 2008. Accra, Ghana: Ghana Health Service (GHS) and ICF Macro.

[7] Ghana Statistical Service (GSS), Ghana Health Service (GHS) & ICF International (2015) Demographic and Health Survey 2014. Rockville, MD: GSS, GHS, and ICF International.

[8] World Health Organization (2007) *Protein and Amino Acid Requirements in Human Nutrition. Report of a Joint WHO/FAO/UNU Expert Consultation*. *WHO Technical Report Series* no. 935. Geneva: WHO.

[9] Ghosh SA, Suri D & Uauy R (2012) Assessment of protein adequacy in developing countries: quality matters. Br J Nutr 108, S77–S87. 23107551 10.1017/S0007114512002577

[10] Ghosh S, Suri D, Vuvor F, (2010) Dietary protein quality is associated with risk of being stunted in peri-urban children in Greater Accra. Poster abstract presented at 2nd World Public Health Congress on Nutrition, October 2010, Porto, Portugal.

[11] Grillenberger M, Neumann CG, Murphy SP, (2003) Food supplements have a positive impact on weight gain and the addition of animal source foods increases lean body mass of Kenyan schoolchildren. J Nutr 133, 3957S–3964S. 14672296 10.1093/jn/133.11.3957S

[12] Hoppe C, Molgaard C, Thomsen BL, (2004) Protein intake at 9 mo of age is associated with body size but not with body fat in 10-y-old Danish children. Am J Clin Nutr 79, 494–501. 14985227 10.1093/ajcn/79.3.494

[13] Michaelsen K, Hoppe C & Mølgaard C (2003) Effect of early protein intake on linear growth velocity and development of adiposity. Monatsschr Kinderheilkd 151, S78–S83.

[14] Hussain T, Abbas S, Khan MA, (2004) Lysine fortification of wheat flour improves selected indices of the nutritional status of predominantly cereal-eating families in Pakistan. Food Nutr Bull 25, 114–122. 15214256 10.1177/156482650402500202

[15] Ghosh S, Smriga M, Vuvor F, (2010) Effect of lysine supplementation on health and morbidity in subjects belonging to poor peri-urban households in Accra, Ghana. Am J Clin Nutr 92, 928–939. 20720257 10.3945/ajcn.2009.28834

[16] Ghosh S, Pellett PL, Aw-Hassan A, (2008) Impact of lysine-fortified wheat flour on morbidity and immunologic variables among members of rural families in northwest Syria. Food Nutr Bull 29, 163–171. 18947029 10.1177/156482650802900302

[17] Semba RD, Shardell M, Sakr Ashour FA, (2016) Child stunting is associated with low circulating essential amino acids. EBioMedicine 6, 246–252. 27211567 10.1016/j.ebiom.2016.02.030 PMC4856740

[18] van Vught AJ, Heitmann BL, Nieuwenhuizen AG, (2010) Association between intake of dietary protein and 3-year-change in body growth among normal and overweight 6-year-old boys and girls (CoSCIS). Public Health Nutr 13, 647–653. 19758483 10.1017/S1368980009991510

[19] Ghosh S, Tano-Debrah K, Aaron GJ, (2014) Improving complementary feeding in Ghana: reaching the vulnerable through innovative business––the case of KOKO Plus. Ann N Y Acad Sci 1331, 76–89. 25514865 10.1111/nyas.12596

[20] Suri DJ, Tano-Debrah K & Ghosh SA (2014) Optimization of the nutrient content and protein quality of cereal:legume blends for use as complementary foods in Ghana. Food Nutr Bull 35, 372–381. 25902596 10.1177/156482651403500309

[21] Ghosh S, Tano-Debrah K, Aaron GJ, (2014) Improving complementary feeding in Ghana: reaching the vulnerable through innovative business—the case of KOKO Plus. Ann N Y Acad Sci 1331, 76–89. 25514865 10.1111/nyas.12596

[22] Lutter CK & Dewey KG (2003) Proposed nutrient composition for fortified complementary foods. J Nutr 133, 3011S–3020S. 12949402 10.1093/jn/133.9.3011S

[23] Food and Agriculture Organization of the United Nations & World Health Organization (2005) *Vitamin and Mineral Requirements in Human Nutrition* https://apps.who.int/iris/bitstream/handle/10665/42716/9241546123.pdf?ua=1

[24] Adu-Afarwuah S, Lartey A, Brown KH, (2007) Randomized comparison of 3 types of micronutrient supplements for home fortification of complementary foods in Ghana: effects on growth and motor development. Am J Clin Nutr 86, 412–420. 17684213 10.1093/ajcn/86.2.412

[25] Fenn B, Morris SS & Frost C (2007) Do childhood growth indicators in developing countries cluster? Implications for intervention strategies. Public Health Nutr 7, 829–834. 10.1079/phn2004632 15482606

[26] Ghana Health Service (GHS) (2013) Good Life: Live it Well. Accra, Ghana: Ghana Health Service.

[27] World Health Organization (2011) *Haemoglobin Concentrations for the Diagnosis of Anaemia and Assessment of Severity. Vitamin and Mineral Nutrition Information System* (WHO/NMH/NHD/MNM/111). https://apps.who.int/iris/bitstream/handle/10665/85839/WHO_NMH_NHD_MNM_11.1_eng.pdf?ua=1

[28] World Health Organization & UNICEF (2009) WHO Child Growth Standards and the Identification of Severe Acute Malnutrition in Infants and Children: A Joint Statement by the World Health Organization and the United Nations Children's Fund. Geneva: WHO. 24809116

[29] Onis M (2006) WHO Motor Development Study: windows of achievement for six gross motor development milestones. Acta Paediatr 95, 86–95. 16817682 10.1111/j.1651-2227.2006.tb02379.x

[30] Lamhaut L, Apriotesei R, Combes X, (2011) Comparison of the accuracy of noninvasive hemoglobin monitoring by spectrophotometry (SpHb) and HemoCue® with automated laboratory hemoglobin measurement. Anesthesiologists 115, 548–554. 10.1097/ALN.0b013e3182270c22 21716091

[31] Takehana S, Yoshida H, Ozawa S, (2016) The effects of pre-analysis sample handling on human plasma amino acid concentrations. Clin Chim Acta 455, 68–74. 26828529 10.1016/j.cca.2016.01.026

[32] Erhardt JG, Estes JE, Pfeiffer CM, (2004) Combined measurement of ferritin, soluble transferrin receptor, retinol binding protein, and C-reactive protein by an inexpensive, sensitive, and simple sandwich enzyme-linked immunosorbent assay technique. J Nutr 134, 3127–3132. 15514286 10.1093/jn/134.11.3127

[33] Leroy J (2011) ZSCORE06: Stata Module to Calculate Anthropometric Z-Scores Using the 2006 WHO Child Growth Standards *[SS Components, editor]*. Boston, MA: Boston College Department of Economics.

[34] Coates J, Swindale A & Bilinsky P (2007) Household Food Insecurity Access Scale (HFIAS) for Measurement of Food Access: Indicator Guide. Washington, DC: FANTA.

[35] Food and Agriculture Organization of the United Nation & FHI 360 (2016) Minimum Dietary Diversity for Women: A Guide for Measurement. Rome: FAO.

[36] Thurnham DI, Mburu AS, Mwaniki DL, (2005) Micronutrients in childhood and the influence of subclinical inflammation. Proc Nutr Soc 64, 502–509. 16313694 10.1079/pns2005468

[37] Suchdev PS, Williams AM, Mei Z, (2017) Assessment of iron status in settings of inflammation: challenges and potential approaches. Am J Clin Nutr 106, 1626S–1633S. 29070567 10.3945/ajcn.117.155937 PMC5701714

[38] Northrop-Clewes CA (2008) Interpreting indicators of iron status during an acute phase response – lessons from malaria and human immunodeficiency virus. Ann Clin Biochem 45, 18–32. 18275670 10.1258/acb.2007.007167

[39] Thurnham DI, McCabe LD, Haldar S, (2010) Adjusting plasma ferritin concentrations to remove the effects of subclinical inflammation in the assessment of iron deficiency: a meta-analysis. Am J Clin Nutr 92, 546–555. 20610634 10.3945/ajcn.2010.29284

[40] Vitamin and Mineral Nutrition Information System (VMNIS) (2014) *C-reactive *P*rotein *C*oncentrations as *a M*arker of *I*nflammation or *I*nfection for *I*nterpreting *B*iomarkers of *M*icronutrient *S*tatus*. Geneva: WHO.

[41] Ballin A, Senecky Y, Rubinstein U, (2012) Anemia associated with acute infection in children. Isr Med Assoc J 14, 484–487. 22977967

[42] Ghosh K & Ghosh K (2007) Pathogenesis of anemia in malaria: a concise review. Parasitol Res 101, 1463–1469. 17874326 10.1007/s00436-007-0742-1

[43] Johnston BC & Guyatt GH (2016) Best (but oft-forgotten) practices: intention-to-treat, treatment adherence, and missing participant outcome data in the nutrition literature. Am J Clin Nutr 104, 1197–1201. 27733397 10.3945/ajcn.115.123315

[44] Dewey KG & Adu-Afarwuah S (2008) Systematic review of the efficacy and effectiveness of complementary feeding interventions in developing countries. Matern Child Nutr 4, 24–85. 18289157 10.1111/j.1740-8709.2007.00124.x PMC6860813

[45] Imdad A, Yakoob MY & Bhutta ZA (2011) Impact of maternal education about complementary feeding and provision of complementary foods on child growth in developing countries. BMC Public Health 11, 14. 21501443 10.1186/1471-2458-11-S3-S25 PMC3231899

[46] Lassi ZS, Das JK, Zahid G, (2013) Impact of education and provision of complementary feeding on growth and morbidity in children less than 2 years of age in developing countries: a systematic review. BMC Public Health 13, 1. 24564534 10.1186/1471-2458-13-S3-S13 PMC3847349

[47] Sales MC, Queiroz EO & Paiva AA (2011) Association between anemia and subclinical infection in children in Paraíba State, Brazil. Rev Bras Hematol Hemoter 33, 96–99. 23284254 10.5581/1516-8484.20110027 PMC3520631

[48] Tanner L, Näntö-Salonen K, Niinikoski H, (2006) Hazards associated with pregnancies and deliveries in lysinuric protein intolerance. Metabolism 55, 224–231. 16423630 10.1016/j.metabol.2005.08.016

[49] Christian P, Shaikh S, Shamim AA, (2015) Effect of fortified complementary food supplementation on child growth in rural Bangladesh: a cluster-randomized trial. Int J Epidemiol 44, 1862–1876. 26275453 10.1093/ije/dyv155 PMC4689999

[50] Phuka JC, Maleta K, Thakwalakwa C, (2008) Complementary feeding with fortified spread is likely to reduce the incidence of severe stunting among 6–18 month old rural Malawian infants. Arch Pediatr Adolesc Med 162, 619–626. 18606932 10.1001/archpedi.162.7.619 PMC3721756

[51] Food and Agriculture Organization of the United Nations, World Health Organization & UNU (2001) Human Energy Requirements. Food and Nutrition Technical Report Series. Rome: FAO.

[52] Batra P, Schlossman N, Balan I, (2016) A randomized controlled trial offering higher-compared with lower-dairy second meals daily in preschools in Guinea-Bissau demonstrates an attendance-dependent increase in weight gain for both meal types and an increase in mid-upper arm circumference for the higher-dairy meal. J Nutr 146, 124–132. 26609172 10.3945/jn.115.218917

[53] Killilea DW, Rohner F, Ghosh S, (2017) Identification of a hemolysis threshold that increases plasma and serum zinc concentration. J Nutr 147, 1218–1225. 28490675 10.3945/jn.116.247171 PMC5443468

