# Supplementary material for: A macro- and micronutrient-fortified complementary food supplement reduced acute infection, improved haemoglobin and showed a dose–response effect in improving linear growth: a 12-month cluster randomised trial
Source: J Nutr Sci. 2019 Jun 27;8:e22. doi: 10.1017/jns.2019.18 (PMC6598226; doi:10.1017/jns.2019.18)
Supplement: Supplementary file 1 [file S2048679019000181sup001.docx]

**Supplementary material**

*

*

*

*

**Supplementary Fig. S1.** Predicted mean length-for-age z-scores at endline in Koko Plus and Micronutrient groups, modelling different levels of supplement consumption^1^

^1^ Mixed effects linear model adjusted for fixed effects of baseline LAZ and mother’s height, and random effects of study cluster and subject to account for repeated measures.

**Supplementary material**

*

**Supplementary Fig. S2.** Predicted mean serum Hb at endline in Koko Plus and Micronutrient groups modelling different levels of supplement consumption ^1^

^1^ Mixed effects linear model adjusted for fixed effects of baseline hemoglobin and random effects of study cluster and subject to account for repeated measures.

**Supplementary material**

*

*

**Supplementary Fig. S3.** Predicted mean serum zinc modelling at endline in Koko Plus and Micronutrient groups modelling different levels of supplement consumption ^1^

^1^ Mixed effects linear model adjusted for fixed effects of baseline serum zinc and random effects of study cluster and subject to account for repeated measures.
